# Epidemiology and Causes of Primary Adrenal Insufficiency in Children: A Population-Based Study

**DOI:** 10.1210/clinem/dgad283

**Published:** 2023-05-22

**Authors:** Joonatan Borchers, Eero Pukkala, Outi Mäkitie, Saila Laakso

**Affiliations:** Children's Hospital, Pediatric Research Center, University of Helsinki and Helsinki University Hospital, Helsinki, Finland; Folkhälsan Institute of Genetics, Folkhälsan Research Center, Helsinki, Finland; Research Program for Clinical and Molecular Metabolism, Faculty of Medicine, University of Helsinki, Helsinki, Finland; Faculty of Social Sciences, Tampere University, Tampere, Finland; Finnish Cancer Registry—Institute for Statistical and Epidemiological Cancer Research, Helsinki, Finland; Children's Hospital, Pediatric Research Center, University of Helsinki and Helsinki University Hospital, Helsinki, Finland; Folkhälsan Institute of Genetics, Folkhälsan Research Center, Helsinki, Finland; Research Program for Clinical and Molecular Metabolism, Faculty of Medicine, University of Helsinki, Helsinki, Finland; Department of Molecular Medicine and Surgery, Karolinska Institutet, and Clinical Genetics, Karolinska University Hospital, Stockholm, Sweden; Children's Hospital, Pediatric Research Center, University of Helsinki and Helsinki University Hospital, Helsinki, Finland; Folkhälsan Institute of Genetics, Folkhälsan Research Center, Helsinki, Finland; Research Program for Clinical and Molecular Metabolism, Faculty of Medicine, University of Helsinki, Helsinki, Finland

**Keywords:** PAI, incidence, prevalence, autoimmune, congenital adrenal hyperplasia

## Abstract

**Context:**

Incidence and causes of primary adrenal insufficiency (PAI) have not been comprehensively studied in children.

**Objective:**

Our objective was to describe the epidemiology and to assess causes of PAI in Finnish children.

**Methods:**

A population-based descriptive study of PAI in Finnish patients aged 0-20 years.

Diagnoses referring to adrenal insufficiency in children born in 1996-2016 were collected from the Finnish National Care Register for Health Care. Patients with PAI were identified by studying patient records. Incidence rates were calculated in relation to person-years in the Finnish population of same age.

**Results:**

Of the 97 patients with PAI, 36% were female. The incidence of PAI was highest during the first year of life (in females 2.7 and in males 4.0/100 000 person-years). At 1-15 years of age, the incidence of PAI in females was 0.3/100 000 and in males 0.6/100 000 person-years. Cumulative incidence was 10/100 000 persons at age of 15 years and 13/100 000 at 20 years. Congenital adrenal hyperplasia was the cause in 57% of all patients and in 88% of patients diagnosed before age of 1 year. Other causes among the 97 patients included autoimmune disease (29%), adrenoleukodystrophy (6%), and other genetic causes (6%). From the age of 5 years, most of the new cases of PAI were due to autoimmune disease.

**Conclusion:**

After the first-year peak, the incidence of PAI is relatively constant through ages 1-15 years, and 1 out of 10 000 children are diagnosed with PAI before the age of 15 years.

Primary adrenal insufficiency (PAI), also called Addison disease, is a rare, potentially life-threatening condition affecting both children and adults. It is defined as insufficient production or action of glucocorticoids, often combined with insufficiency of mineralocorticoids, caused by destruction or dysfunction of adrenal cortex (1). Patients with PAI require lifelong treatment with glucocorticoid replacement, often combined with mineralocorticoid replacement.

In adults, the most common cause of PAI is autoimmune disease. In contrast, in children from 50% up to 86% of PAI are due to congenital adrenal hyperplasia (CAH), mostly due to 21-hydroxylase deficiency (2-4). According to newborn screenings, the prevalence of CAH varies between 1/10 000 and 1/20 000 births (5, 6). In addition to the more severe forms of CAH, these screenings enable detection of the vast majority of milder forms of CAH in newborns (7).

The incidence and causes of PAI in childhood remain scarcely studied. Two single-center studies from North America and 1 multicenter study from Italy reported autoimmune disease as the second most common cause (2-4). Autoimmune PAI may present as an isolated disease or as a manifestation of a polyendocrinopathy (8-10). As part of a polyendocrinopathy, PAI is a common manifestation of autoimmune polyendocrinopathy–candidiasis–ectodermal dystrophy (APECED), also known as autoimmune polyendocrine syndrome type 1 (APS-1). In patients with APECED, PAI is diagnosed in 55% to 84% of all patients in different cohorts, and the variation in prevalence may partly be explained by variable length of follow-up (11-16). In patients with APECED, the age at diagnosis of PAI ranges from 2 to 55 years (11-15). PAI is also a major manifestation of autoimmune polyendocrine syndrome type 2 (APS-2). Although the mean age at diagnosis of PAI in APS-2 has been reported to be 35 years, some patients with APS-2 have already developed PAI in childhood (2, 3, 17, 18).

In 2 single-center studies from Australia and China other non-CAH causes seem to be as common as or more common than autoimmune disease (19, 20). Other causes of childhood PAI include adrenal hemorrhage, X-linked adrenoleukodystrophy (XALD), and other rare genetic disorders (2, 3, 21). The prevalence of XALD in newborn males is estimated to be approximately 1/20 000 and approximately 80% of males with XALD develop PAI (22, 23). Other genetic causes of PAI, mostly monogenic disorders, are very rare (21). These rare genetic defects may explain most of the cases of PAI with previously unknown cause (24-26).

The disease etiology has important implications for treatment and follow-up of PAI. As the epidemiology and causes of PAI in children remain incompletely studied, we aimed to determine the population-based incidence and causes of PAI in Finnish children. By using national registries, we were able to explore the nationwide incidence and characteristics of the disease in all children and adolescents diagnosed with PAI and treated or followed in any Finnish hospital.

## Materials and Methods

### Subjects

To identify all Finnish patients diagnosed with PAI aged 0-20 years we used the Finnish National Care Register for Health Care, which includes diagnoses registered in any Finnish hospital. We collected data of all patients born and diagnosed in 1996-2016 with the following diagnosis codes, according to the 10th revision of the International Classification of Diseases (ICD-10): E25.0, E25.00, E25.01, E25.8, E25.9, E27.1, E27.4, E27.8, E27.9, A18.7, E35.1, E35.1*A39.1, Q89.1. The study was approved by the Research Ethics Committee of the Hospital District of Helsinki and Uusimaa. No patient consents were required as the study was solely based on registry data.

**Figure 1.**
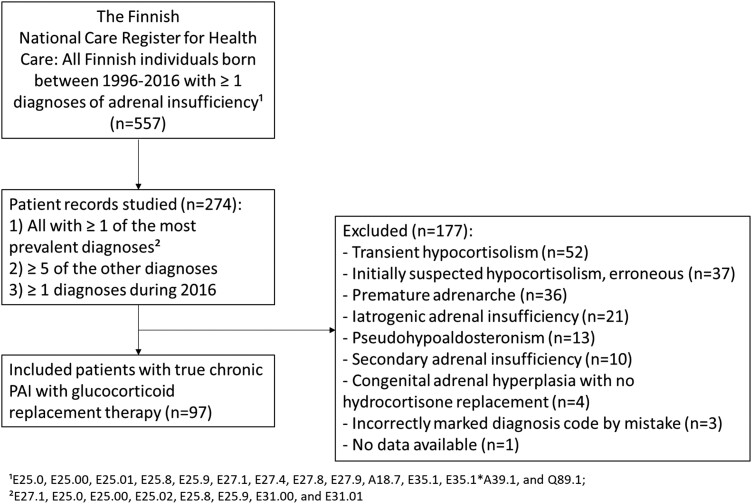
Identification of patients diagnosed with primary adrenal insufficiency (PAI) during 1996-2016 before the age of 21 years in Finland.

Altogether 557 individuals had received 1 of the included diagnoses at least once by the end of 2016 (Fig. 1). We excluded all patients with transient adrenal insufficiency and patients with CAH who did not require glucocorticoid replacement therapy. To identify patients with PAI, patient records were reviewed from the patients with 1 or more diagnoses of PAI, CAH, or polyendocrinopathy (E27.1, E25.0, E25.00, E25.02, E25.8, E25.9, E31.00, and E31.01). In order to not miss any patients with PAI, we also reviewed the records of the patients who had 5 or more of the other diagnoses of unspecified adrenal insufficiency, unspecified adrenal disorder, adrenal insufficiency due to adrenal infection, or adrenal malformation (E27.4, E27.8, E27.9, A18.7, E35.1, E35.1*A39.1, Q89.1). In addition, we studied the patient records of all individuals who had 1 or more of any of the diagnoses during 2016 so that we did not miss any patients with PAI who may have been diagnosed toward the end of the follow-up period. According to these criteria, we studied the patient records of 274 patients. To confirm that our criteria accurately identify all patients with PAI, from 1 hospital district we additionally reviewed the patient records of all individuals who had received at least once any of the above-mentioned diagnoses. In total, we found 54 patients who had received any of the following diagnoses fewer than 5 times: E25.0, E25.00, E25.01, E25.8, E25.9, E27.1, E27.4, E27.8, E27.9, A18.7, E35.1, E35.1*A39.1, or Q89.1. None of these patients had PAI.

The follow-up ended at the end of 2016 or at death, whichever was first. The diagnosis of PAI was based on the following criteria: (1) serum cortisol below the age- and method-specified reference range combined with increased level of adrenocorticotropic hormone (ACTH) with no recovery in the values during the follow-up or (2) the result of ACTH-stimulation test below the reference range, and (3) long-term treatment with glucocorticoid replacement therapy with or without mineralocorticoid treatment was required for inclusion.

The diagnosis of autoimmune PAI was based on positive adrenal autoantibodies. For 2 patients, information on adrenal autoantibodies was not available but PAI was considered to be autoimmune since in addition to PAI both had type 1 diabetes and 1 also had hypothyroidism. The diagnosis of CAH was based on the increased level of 17-hydroxyprogesterone and/or known defect in CAH-causing genes. All cases of secondary or iatrogenic adrenal insufficiency were excluded. Altogether 97 of the 274 patients fulfilled the diagnostic criteria of PAI (Fig. 1).

For the 97 verified patients with PAI, we collected the following parameters from the patient records: age at diagnosis of PAI, age at initiation of glucocorticoid and mineralocorticoid treatment, other diagnosed autoimmune diseases, and age at death. Results of molecular genetic examination were collected if available.

### Statistics

For the incidence calculation, we extracted population data for persons born in Finland in 1996-2016 from Statistics Finland. We calculated age-specific annual incidence rates by dividing the number of diagnosed patients with PAI at each age year by the total Finnish population of the same age. Cumulative incidence was calculated by summing 1-year age-specific incidence rates. Because overall mortality in children is low, cumulative incidence at a given age can be interpreted as an approximation of prevalence at that age. As the number of the subjects born in 1996-2016 who had reached the age of 15 years by the end of 2016 was small, we mainly report incidence rates up to the age of 15.

## Results

After exclusion of all cases with transient glucocorticoid deficiency and CAH with no glucocorticoid replacement, 97 patients diagnosed with PAI before age 21 years remained. Of the total cohort of 97 patients, 35 (36%) were female. The median age at diagnosis was 3 years (range 0.0-19.6 years). Two patients died during the follow-up. One patient died at 10 years of age for severe XALD, and 1 patient died at 17 years of age for an unknown syndrome with ACTH resistance.

### Incidence and Prevalence of PAI

**Figure 2.**
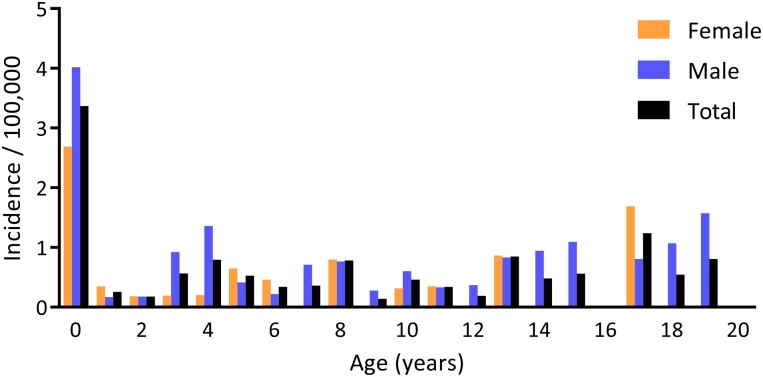
Age-specific incidence of primary adrenal insufficiency in Finland per 100 000 person-years for ages 0-20 years, 1996-2016, by sex.

The highest age-specific incidence of PAI was observed in the population younger than 1 year of age: 3.4/100 000 person-years (2.7/100 000 in females and 4.0/100 000 in males) (Fig. 2). The incidence in ages 1-15 years was markedly lower: 0.4/100 000 (0.3/100 000 in females and 0.6/100 000 in males).

The incidence of CAH before the age of 1 year was 3.0/100 000 person-years in all patients, 2.5/100 000 person-years in females and 3.4/100 000 person-years in males. When CAH was excluded, the incidence for non-CAH PAI was before 1 year of age 0.4/100 000 person-years in all patients, 0.2/100 000 person-years in females and 0.6/100 000 person-years in males. The incidence of all non-CAH patients between 1 and 15 years of age was 0.3/100 000 person-years, in females 0.2/100 000, and in males 0.3/100 000. The highest age-specific annual incidence in non-CAH patients between the ages of 0 and 15 years was found at the age of 13 years (0.8/100 000 person-years). The highest age-specific incidence of non-CAH patients between 0 and 15 years of age was found for females at the age of 13 (0.9/100 000 person-years) and for males at the age of 15 (1.1/100 000 person-years).

**Figure 3.**
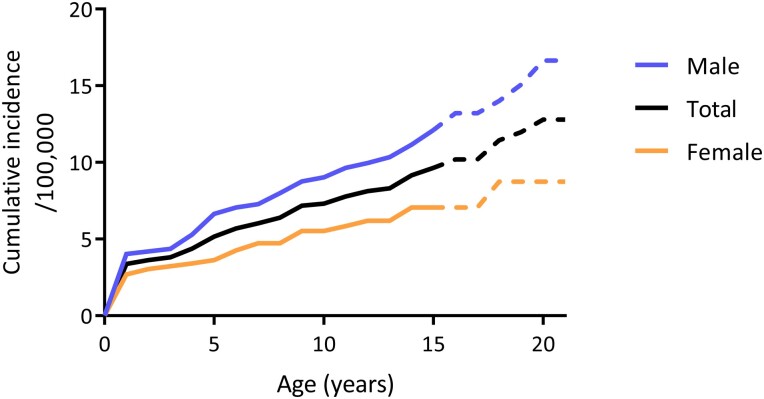
Cumulative incidence of PAI in Finland up to 20 years of age in total and in males and females separately. The dashed line represents the estimation of cumulative incidence between 15 and 20 years of age.

The incidence of PAI was highest during the first year of life, and from 1 year onwards the cumulative incidence increased constantly throughout the entire childhood (Fig. 3). The cumulative incidence of PAI at 15 years of age was 10/100 000 persons (7/100 000 females, 13/100 000 males) and at 20 years of age 13/100 000 persons (9/100 000 females, 17/100 000 males).

### Causes of PAI

CAH was the cause of PAI in 55 (57%) patients. Of the 55 patients with CAH, 36 (12/36, 33% female) were reported to have a defect in *CYP21A2* and 1 male patient in *HSD3B2*; the genetic screening result was unavailable in 18 (7/18, 39% female) patients. The second most common cause of PAI was autoimmune disease with 28 (29%) patients. XALD was found in 6 (6%) patients. Other rare causes were found in 6 patients in total. These included genetic causes in 5 patients: 1 patient with adrenal hypoplasia due to a defect in *NR0B1*, 2 patients with ACTH resistance due to a defect in *MRAP*, 1 patient with triple A syndrome due to a defect in *AAAS*, and 1 patient with Mulibrey nanism due to a defect in *TRIM37.* In addition, 1 patient had been diagnosed with adrenal aplasia during fetal ultrasound. This patient's sibling had died soon after birth and adrenal aplasia was detected in the autopsy, but no genetic testing was performed in either of the siblings. In addition, 2 patients had PAI of an unknown etiology (Table 1).

**Table 1.** Causes of PAI in the 97 Finnish children and adolescents aged 0-20 years with PAI diagnosed during 1996-2016

Cause	Female	Male	Total
Congenital adrenal hyperplasia	20	35	55
Autoimmune	12	16	28
Isolated	4	9	13
Syndromic	8	7	15
X-linked adrenoleukodystrophy	0	6	6
Other known cause	2	4	6
Adrenal aplasia	0	1	1
Adrenal hypoplasia*^a^*	0	1	1
ACTH resistance*^b^*	1	1	2
Triple A syndrome*^c^*	0	1	1
Mulibrey nanism*^d^*	1	0	1
Unknown	1	1	2

Abbreviation: ACTH, adrenocorticotropic hormone; PAI, primary adrenal insufficiency.

Defect in *NR0B1* gene.

Defect in *MRAP* gene.

Defect in *AAAS* gene.

Defect in *TRIM37* gene.

Of the patients with CAH, 65% were diagnosed before the age of 1 year (mean age at diagnosis 1.5; range 0.0-8.1); 75% of female patients with CAH (mean age at diagnosis 1.0; range 0.0-8.1) and 60% of males (mean age at diagnosis 1.8; range 0.0-7.3, *P* = .017) were diagnosed before the age of 1 year. Three male patients were found via the newborn screening for metabolic diseases that was launched in Finland in 2015. For all non-CAH patients, the median age at diagnosis was 8 years (range 0.0-19.6). For patients with autoimmune PAI, the median age at diagnosis was 11 years for both patients with isolated autoimmune PAI (range 5.7-18.7) and patients with autoimmune polyendocrine syndrome (range 2.5-19.6). For characteristics of all causes, see Table 2.

**Table 2.** Characteristics of patients diagnosed with PAI at the age of 0-20 years during 1996-2016 in Finland, by cause of PAI. Percentages refer to the proportion of females.

Characteristic	Cause					
	CAH	Autoimmune	XALD	Other	Unknown	All
Number (female %)	55 (36%)	28 (43%)	6 (0%)	6 (33%)	2 (50%)	97 (36%)
Age at diagnosis, median (range)	0.1 (0.0-8.1)	11.3 (2.5-19.6)	8.4 (3.2-13.1)	0.4 (0.0-6.1)	2.3 (0.5-4.1)	3.3 (0.0-19.6)
Age at initiation of GC, median (range)	0.1 (0.0-8.1)	11.3 (3.7-19.5)	8.8 (3.2-12.9)	0.3 (0.0-6.1)	2.3 (0.5-4.1)	3.3 (0.0-19.5)
Age at initiation of MC, median (range)	0.1 (0.0-16.6)*^a^*	11.3 (2.5-19.6)	8.7*^b^*	0.3 (0.0-10.8)*^c^*	9.4 (7.3-11.5)	3.3 (0.0-19.6)

Abbreviations: CAH, congenital adrenal hyperplasia; GC, glucocorticoid; MC, mineralocorticoid; Other, other known cause; PAI, primary adrenal insufficiency; XALD, X-linked adrenoleukodystrophy;

n = 54; *^b^*n = 1; *^c^*n = 4.

**Figure 4.**
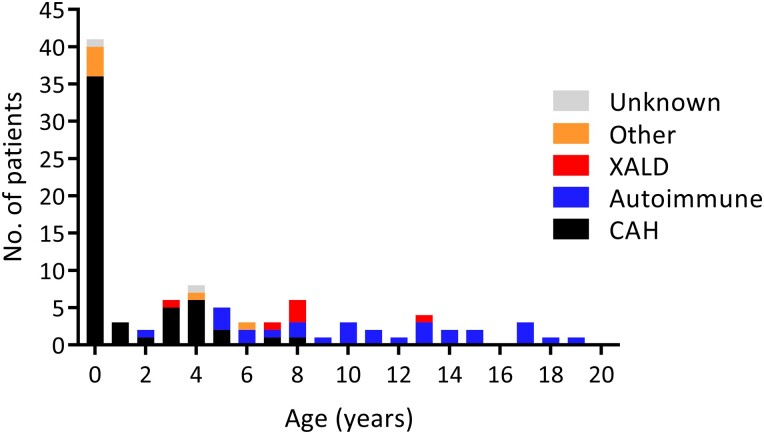
Number of patients aged 0-20 years diagnosed with primary adrenal insufficiency according to cause and age during 1996-2016. Other, other known cause; XALD, X-linked adrenoleukodystrophy; CAH, congenital adrenal hyperplasia.

In patients younger than 1 year of age (n = 41), CAH was the cause of PAI in 36 (88%) patients (Fig. 4). The 5 non-CAH patients younger than 1 year had a known genetic disorder (3 patients), adrenal aplasia (1 patient), and PAI of unknown cause (1 patient). In the other age groups, the cause of PAI was CAH in 19 (34%) patients, autoimmune in 28 (50%) patients, XALD in 6 (11%) patients, other known cause in 2 (4%) patients, and unknown etiology in 1 patient. CAH was the most common cause of PAI until 4 years of age. From 5 years onwards the proportion of autoimmune disease increased and from 8 years onwards 95% of new cases were due to autoimmune disease (Fig. 4).

Of the patients with PAI due to an autoimmune process, 8 (29%) had been diagnosed with APECED. All but 1 patient with APECED had been found to carry 2 known pathogenic variants of the *AIRE* gene and the remaining patient had 1 known pathogenic variant. In 12 of the remaining 20 patients with autoimmune disease, the Finnish major mutation of *AIRE* was excluded. Of these 20 patients with autoimmune PAI, 7 (35%) had other autoimmune diseases and were classified as APS-2. Of these 7 patients, 3 had type 1 diabetes mellitus, 1 had autoimmune hypothyroidism, 2 had both type 1 diabetes mellitus and hypothyroidism, and 1 had autoimmune hypogonadism. The remaining 13 patients with isolated autoimmune PAI had not developed any other autoimmune diseases by the end of follow-up at the age of 5.7-18.7 years (Table 1).

## Discussion

In this population-based study we report the epidemiology and the spectrum of causes in all known child and adolescent patients with PAI in Finland during 1996-2016.

In our study, we found that the incidence of PAI during the first year of life is higher than later in childhood, and from age 1 year onwards new cases of PAI are diagnosed quite evenly throughout the childhood. We found that the prevalence (cumulative incidence) of PAI at 15 years of age was 10 cases/100 000, and the estimation of prevalence at 20 years of age was 13 cases/100 000. The prevalence of PAI including both children and adults has been reported in several studies from different populations in Europe. In 2 studies from different areas of England the prevalence was 9.3 and 11/100 000, in Italy 12/100 000, in Norway 14/100 000, and in Sweden 13/100 000 (10, 27-31). The highest reported prevalence of PAI is from Iceland, where the prevalence in 2016 was 22/100 000. The incidence of PAI in Europe has been reported to vary between 0.4 and 0.6/100 000 person-years (10, 28, 30, 31). Reports from outside Europe are scarce but the prevalence of PAI seems to be lower, for instance, in Korea (0.4/100 000) (32). However, it is unclear whether all the reports mentioned above include patients with CAH. The prevalence of PAI in childhood has not been comprehensively reported previously.

Our findings indicate that in Finland PAI is more prevalent in males than in females during childhood. In non-CAH patients, this was partly due to XALD, which was found only in males. However, autoimmune PAI and rare genetic causes were also found more often in males than females even though autoimmune PAI is typically more prevalent in females, at least in adults (10, 27, 28). A male predominance of non-CAH PAI was reported in an Italian study that showed a higher number of males with XALD and rare monogenic causes, some of which were rare causes of CAH (4). No male predominance was found in patients with autoimmune PAI in Italy. However, in the scarce reports on autoimmune PAI in children the findings vary. In a US study on PAI, 62% of children with autoimmune PAI were males (2) whereas in a Canadian study 38% of patients at any age with autoimmune PAI were males (3). In a Swedish study of PAI due to probable autoimmune cause, the mean age at diagnosis of males was 35.4 years and 42.2 years for females (31). Thus, it may be that autoimmune PAI is not more common in females during childhood, but the proportion of affected females increases later in adulthood. Further studies are warranted to analyze whether our observation of male predominance among patients with autoimmune PAI is due to genetic characteristics of the Finnish population, due to other still unknown factors, or due to chance.

Our study showed a clear difference between the sexes in the number of patients with CAH. CAH was more prevalent in males both in patients with and without genetic screening results available. The difference between sexes is notable since considering the autosomal recessive inheritance pattern, CAH should be found evenly between males and females. In the Italian study, rare etiology of CAH was diagnosed more often in males than in females but the sex ratio for CAH due to *CYP21A2* defects was not reported (4). On the other hand, previous studies on newborn screening for CAH have not found a clear predominance of either sex (5, 7, 33). In Finland, newborn screenings for metabolic diseases were launched in 2015, and 3 male patients in our cohort were found via screening. No female patients were diagnosed via newborn screening in Finland during 2015-2016. In previous studies on Finnish patients with CAH, conducted in 1997 and 1999, no similar difference between sexes was found in patients who were born and diagnosed mainly before the beginning of our follow-up time (34, 35). It could be speculated that predominance of males diagnosed with CAH could be due to a higher proportion of induced abortions of affected female fetuses. All Finnish families with a child with CAH are referred to genetic counseling according to international guidelines (36), and prenatal diagnostics are available nationwide on requests for CAH families, possibly leading to a higher proportion of induced abortions of virilized female fetuses than of affected male fetuses. The effect of genetic counseling should be further analyzed by studying the sex ratio of affected siblings in families with more than 1 child with CAH. So far, there are no studies on the effects of genetic counseling and prenatal diagnostics on diseases like CAH and our cohort size unfortunately did not allow such an analysis.

In this study we found that in patients aged 0-20 years, CAH was the most prevalent cause for PAI. However, the percentage of PAI due to an autoimmune process increased rapidly after the age of 4 years and it was the second most common cause for PAI in all patients. These findings are in concordance with most of the previous findings (2-4). However, there are some populations where other non-CAH causes including XALD and congenital adrenal hypoplasia seem to be as common or more common than autoimmune disease (19, 20). XALD was found only in male patients with PAI, as expected, and it was the third most common cause for PAI. The percentage of patients with PAI due to XALD varies in different populations and XALD seems often to be a more common cause of PAI than what was found in our data (3, 4, 19, 20, 22, 23). In our study we did not search patients separately for the ICD-10-diagnosis of XALD because we assumed that all patients with XALD who had been diagnosed with PAI had also been registered with a separate PAI diagnosis. It is possible that we have missed some patients with XALD.

In addition to CAH, autoimmune disease, and XALD, we also found 6 patients with other known rare causes and only 2 patients with unknown etiology. All 6 patients with a known rare cause, except 1 with adrenal aplasia, had a previously described monogenic disease (21, 37). A defect in steroidogenic factor-1 (*NR5A1*) and dosage-sensitive sex reversal, adrenal hypoplasia critical region on the chromosome X, gene 1 (DAX1, *NR0B1*) have been reported to cause a defect in the development of adrenal glands and sometimes adrenal agenesis (38, 39). However, these gene defects were not screened in the patient and the sibling with adrenal aplasia in our cohort.

In our cohort, CAH was usually diagnosed during the first year of life and a minority of cases were diagnosed after 4 years of age. We included only more severe forms of CAH that required regular treatment with glucocorticoids. In these patients, CAH affected 1/33 000 children during the first year of life, which is lower than the estimated incidence of CAH worldwide (5, 6). However, the previous estimations are based on newborn screenings, which also detect most of the mildest cases of CAH, which in our study were diagnosed after the first year of life or were not all classified as PAI cases. Disease severity and age at onset of symptoms correlate with the degree of defect in the affected enzyme (40, 41). Even though the gene defect is congenital, the disease is often diagnosed weeks or even years after birth, especially in males who do not have signs of virilization in birth. In our cohort, CAH in females was diagnosed at a slightly younger age than in males. It has been shown previously that the majority of cases with classic CAH are diagnosed during the first year of life and usually before the age of 4 years in the remaining patients (42). Among our patients with PAI, the proportion of autoimmune disease increased substantially after 5 years of age, and after 8 years of age, 95% of all new cases of PAI were due to an autoimmune process. It has been shown that an autoimmune process is the most common cause of PAI in adults (30, 43). However, our results indicate that this is also true in children over 5 years of age.

The patients in our cohort were identified from a national registry that includes comprehensive patient data from all hospitals in Finland. The low number of patients limits the power of the study. Due to the specific genetic background in Finland, including increased prevalence of APECED, the results may not be fully generalizable worldwide.

In summary, our population-based data from Finland show that the highest incidence of PAI in children is seen soon after birth when most of the patients with PAI with CAH are diagnosed. After the first-year peak in incidence, the cumulative incidence increases steadily throughout childhood. In addition, in children older than 4 years, newly diagnosed PAI is most likely due to an autoimmune process that had previously been reported as the main cause of PAI in adults. It is important to recognize these differences in PAI etiology in different ages since the cause of PAI affects the diagnostic measures, treatment, and follow-up of the patient (43, 44).

## Data Availability

Restrictions apply to the availability of some or all data generated or analyzed during this study to preserve patient confidentiality or because they were used under license. The corresponding author will on request detail the restrictions and any conditions under which access to some data may be provided.

## Abbreviations

ACTH: adrenocorticotropic hormone
APECED: autoimmune polyendocrinopathy–candidiasis–ectodermal dystrophy
APS: autoimmune polyendocrine syndrome
CAH: congenital adrenal hyperplasia
PAI: primary adrenal insufficiency
XALD: X-linked adrenoleukodystrophy
